# Gene expression profiles in clinically T1-2N0 ER+HER2− breast cancer patients treated with breast-conserving therapy: their added value in case sentinel lymph node biopsy is not performed

**DOI:** 10.1007/s10549-023-07128-2

**Published:** 2023-10-05

**Authors:** L. M. van Roozendaal, M. L. G. Vane, E. Colier, L. J. A. Strobbe, M. de Boer, G. Sonke, M. C. Van Maaren, M. L. Smidt

**Affiliations:** 1 Department of Surgical Oncology, Zuyderland Medical Center, Heerlen - Sittard, The Netherlands; 2 Department of Surgical Oncology, Maastricht University Medical Centre, Maastricht, The Netherlands; 3 GROW – School for Oncology and Developmental Biology, Maastricht University Medical Center, Maastricht, The Netherlands; 4 Department of Surgical Oncology, Canisius-Wilhelmina Hospital, Nijmegen, The Netherlands; 5 Department of Medical Oncology, Maastricht University Medical Center, Maastricht, The Netherlands; 6 Department of Medical Oncology, Netherlands-Cancer Institute, Amsterdam, The Netherlands; 7 Department of Health Technology and Services Research, Technical Medical Centre, University of Twente, Enschede, The Netherlands; 8 Department of Research and Development, Netherlands Comprehensive Cancer Organisation (IKNL), Utrecht, The Netherlands

**Keywords:** Breast cancer, Sentinel lymph node biopsy, Gene expression profile, Adjuvant chemotherapy

## Abstract

**Purpose:**

Omitting sentinel lymph node biopsy (SLNB) in breast cancer treatment results in patients with unknown positive nodal status and potential risk for systemic undertreatment. This study aimed to investigate whether gene expression profiles (GEPs) can lower this risk in cT1-2N0 ER+ HER2– breast cancer patients treated with BCT.

**Methods:**

Patients were included if diagnosed between 2011 and 2017 with cT1-2N0 ER+ HER2– breast cancer, treated with BCT and SLNB, and in whom GEP was applied. Adjuvant chemotherapy recommendations based on clinical risk status (Dutch breast cancer guideline of 2020 versus PREDICT v2.1) with and without knowledge on SLNB outcome were compared to GEP outcome. We examined missing adjuvant chemotherapy indications, and the number of GEPs needed to identify one patient at risk for systemic undertreatment.

**Results:**

Of 3585 patients, 2863 (79.9%) had pN0 and 722 (20.1%) pN + disease. Chemotherapy was recommended in 1354 (37.8% guideline-2020) and 1888 patients (52.7% PREDICT). Eliminating SLNB outcome (*n* = 722) resulted in omission of chemotherapy recommendation in 475 (35.1% guideline-2020) and 412 patients (21.8% PREDICT). GEP revealed genomic high risk in 126 (26.5% guideline-2020) and 82 patients (19.9% PREDICT) in case of omitted chemotherapy recommendation in the absence of SLNB. Extrapolated to the whole group, this concerns 3.5% and 2.3%, respectively, resulting in the need for 28–44 GEPs to identify one patient at risk for systemic undertreatment.

**Conclusion:**

If no SLNB is performed, clinical risk status according to the guideline of 2020 and PREDICT predicts a very low risk for systemic undertreatment. The number of GEPs needed to identify one patient at risk for undertreatment does not justify its standard use.

## Introduction

Three randomized controlled trials (i.e., BOOG 2013-08, SOUND, and INSEMA) investigate on the safety of omitting sentinel lymph node biopsy (SLNB) in cT1-2N0 breast cancer patients treated with breast-conserving therapy (BCT) [1–3]. Recently, presented results of SOUND trial (cT1N0 breast cancer) show non-inferiority for regional recurrence risk and overall survival [4], and INSEMA recently published on quality of life results revealing significantly less morbidity including pain and arm swelling and improved shoulder mobility in the no-SLNB group [5]. Final safety results of all three trials will be published in near future, but assumably, omission of SLNB will be implemented in daily practice leading to absent information on pathological lymph node status in this specific patient population. This information, besides tumor and patient characteristics, is generally used for chemotherapy recommendations. Patients with absent SLNB information and unknown positive nodal status could be at risk for systemic undertreatment, potentially resulting in an increased risk of distant metastases and decreased disease-free and overall survival (OS).

Gene expression profiles (GEPs) were developed to improve the risk assessment of distant metastases in breast cancer patients compared to the risk based on traditional tumor characteristics as adopted in, for example, breast cancer guidelines and the PREDICT tool [6, 7]. PREDICT is an online tool to predict 5- and 10-year overall survival and the expected benefits of systemic therapy for breast cancer patients, based on patient- and tumor characteristics. The 70-gene signature test (Mammaprint®) is an example of a GEP that helps estimate the risk for distant metastases in early-stage breast cancer patients with lymph node-negative disease or 1–3 positive lymph nodes [8, 9]. Another example is the 21-gene Oncotype DX Breast Recurrence Score®, which predicts the 10-year risk of distant metastases in ER + HER2– pN0 breast cancer. Both tests are thereby predictive for the likelihood that chemotherapy is beneficial [10–12]. GEPs could be of help to estimate the risk of distant metastases and the added value of adjuvant chemotherapy in ER + HER2– breast cancer patients in case SLNB is omitted.

This study thereby aims to determine the added value of GEPs in cT1-2N0 ER + HER2– breast cancer patients treated with BCT in whom SLNB could have been omitted.

## Material and methods

### Study population

GEP was applied in a total of 3585 patients aged < 70 years diagnosed between 2011 and 2017 with cT1-2N0 ER + HER− breast cancer and who were treated with BCT and SLNB in the Netherlands. The cN0 status was based on negative physical examination and/or negative axillary ultrasound, or negative cyto- or histology. Data on patient-, tumor-, diagnostic-, and treatment-related characteristics including pathology outcome after lumpectomy and SLNB were retrieved from the Netherlands Cancer Registry, a nationwide population-based registry including all newly diagnosed malignancies since 1989. Isolated tumor cells (< 0.2 mm) in sentinel lymph nodes were recorded as histologically node-negative. Registration on the use of GEPs (Mammaprint® and 21-gene Oncotype DX Breast Recurrence Score®) was performed since 2011. Since guidelines are inconclusive about the benefit of adjuvant chemotherapy in patients aged > 70 years, these patients were excluded. Other exclusion criteria were missing information on GEP outcome or tumor size, tumor grade, receptor status, and SLNB.

### Recommendation for adjuvant chemotherapy in ER+ HER2– tumors

In ER+ HER2– breast cancer patients, the Dutch breast cancer guideline of 2020 recommends adjuvant chemotherapy in patients with grade I breast cancer and tumor size > 3.0 cm, grade II and tumor size > 2.0 cm, or grade III and tumor size > 1.0 cm. In patients aged < 35 years, adjuvant chemotherapy is recommended for grade I breast cancer and tumor size ≥ 2.0 cm, or grade II–III and tumor size > 1.0 cm. Furthermore, adjuvant chemotherapy is recommended in patients with a positive pathological lymph node status, except in case of grade I breast cancer with tumor size ≤ 2.0 cm [13].

In addition to the guideline of 2020, it is recommended to use the online tool PREDICT version 2.1 to guide the decision-making process [6, 7]. Adjuvant chemotherapy using PREDICT v2.1 was considered beneficial in case of an expected absolute 10-year OS gain of at least 3–5% (considered as clinical high risk). In this study, patients with a calculated ≥ 3% 10-year OS gain were considered as clinical high risk. The expected 10-year OS (PREDICT v2.1) is based on patient- and tumor characteristics (i.e., age, menopausal status, invasive tumor size, tumor grade, method of detection (screening/symptoms/or unknown), and number of positive lymph nodes). Ki-67 status was not registered in the Netherlands Cancer Registry and was therefore set to unknown.

The outcome of the 70-gene signature test (Mammaprint®) is reported as genomic low or genomic high risk. The 21-gene Oncotype DX Breast Recurrence Score® ranges from 0 to 100, resulting in low risk (0–10), intermediate risk (11–25), or high risk of recurrence (26–100). According to previous studies, patients in our study with an intermediate risk score based on their 21-gene Oncotype DX Breast Recurrence Score® were previously considered as genomic low risk [10, 12]. Based on current literature, clinical high-risk patients aged 50 or younger with a low or intermediate genomic risk do benefit from chemotherapy [14, 15].

### Statistics

Patient- and tumor characteristics were presented using descriptive statistics. We assessed clinical risk status for each patient based on the guideline of 2020 and PREDICT v2.1, and the GEP outcome was presented. Subsequently, the clinical risk status was assessed without information on SLNB outcome. We compared the clinical risk status to the GEP outcome. Then, we examined the number of patients who would have missed their adjuvant chemotherapy indication in case of absent SLNB information and changed clinical status from high to low risk, in combination with a genomic high-risk score. We calculated the number of GEPs needed to identify one patient at risk for chemotherapy undertreatment. Statistical analyses were performed using the Statistical Package for the Social Sciences (SPSS) version 25.0 (IBM Corporation, Armonk, NY, USA)**.** Differences in categorical variables between the clinical low and high-risk groups are presented as absolute numbers and percentages and compared using the Pearson Chi-squared test. Age as continuous variable is presented as median with interquartile range and compared between clinical low and high-risk groups using the Wilcoxon rank-sum test.

## Results

### Patient and tumor characteristics

GEP was applied in a total of 3,585 cT1-2N0 ER+ HER2– patients aged < 70 who were treated with BCT. Patient and tumor characteristics are summarized in Table 1. Almost two thirds of the patients had a postmenopausal status (63.4%), and invasive carcinoma of no special type (NST) was the most common subtype (86.3%). The sentinel lymph node was negative in 2,863 patients (79.9%), contained micrometastatic disease in 371 patients (10.3%), and one or more macrometastases in 351 patients (9.8%). Completion axillary lymph node dissection (ALND) was performed in 93 patients (2.6%). Final pathology showed a pN0 status in 2,863 (79.9%) and pN + disease in 722 patients (20.1%). Of the pN + patients, 371 (10.3%) had pN1mi disease, 348 (9.7%) pN1, two were staged as pN2 (0.1%) and one as pN3. Adjuvant chemotherapy was administered to 1,120 of 3,585 patients (31.2%).

**Table 1:** Baseline characteristics of cT1-2N0 ER+ HER2− breast cancer patients in whom GEP is performed

Variable	Overall 3585 (100%)	Genomic low risk 2319 (100%)	Genomic high risk 1266 (100%)	*p* value
Age median (IQR) Age category, n (%)	57 (13)	56 (12)	57 (13)	0.247
< 35	12 (0.3)	5 (0.2)		
35–50	746 (20.8)	484 (20.9)	362 (20.6)	
50–70	2827 (78.9)	1830 (78.9)	997 (78.8)	
Menopausal status, n (%)				
Premenopausal	799 (2.3)	538 (23.2)	261 (20.6)	0.033
Perimenopausal	275 (7.7)	187 (8.1)	88 (7.0)	
Postmenopausal	2272 (63.4)	1429 (61.6)	843 (66.6)	
Unknown	239 (6.7)	165 (7.1)	74 (5.8)	
Tumor type, n (%)				
NST	3095 (86.3)	1957 (84.4)	1138 (89.9)	< 0.001
Lobular	284 (8.0)	225 (9.7)	59 (4.6)	
Other	206 (5.7)	371 (5.9)	69 (5.5)	
Pathologic T stage, n (%)				
T1	2854 (79.6)	1874 (80.8)	980 (77.4)	0.053
T2	728 (20.3)	443 (19.1)	285 (22.5)	
T3	3 (0.1)	2 (0.1)	1 (0.1)	
Pathologic N stage, n (%)				
N0	2863 (79.9)	1802 (77.7)	1061 (83.8)	< 0.001
N1mi	371 (10.3)	264 (11.4)	107 (8.5)	
N1	348 (9.7)	252 (10.9)	96 (7.6)	
N2	2 (0.1)	0 (0.0)	2 (0.2)	
N3	1 (0.0)	1 (0.0)	0 (0.0)	
Completion ALND performed, n (%)				
Yes	93 (2.6)	56 (2.4)	37 (2.9)	0.361
No	3492 (97.4)	2263 (97.6)	1229 (97.1)	
PR status, n (%)				
Positive	3037 (84.7)	2065 (89.0)	972 (76.8)	< 0.001
Negative	548 (15.3)	254 (11.0)	294 (23.2)	
Tumor grade, n (%)				
I	539 (15.0)	454 (19.6)	85 (6.7)	< 0.001
II	2496 (69.7)	1708 (73.6)	788 (62.3)	
III	550 (15.3)	157 (6.8)	393 (31.0)	

P-values were calculated using Pearson Chi-squared tests or Wilcoxon rank-sum tests

*IQR* interquartile range. *GEP* gene expression profile, *N (%)* number of cases, *SD* standard deviation, *NST* invasive carcinoma of no specific type, *T* tumor stage, *N* lymph node stage, *N1mic* micro metastasis, *ALND* axillary lymph node dissection, *PR* progesterone receptor

### Clinical risk status with and without SLNB information

According to the Dutch breast cancer guideline of 2020, 2231 patients (62.2%) were marked as clinical low risk and 1354 patients (37.8%) as clinical high risk. According to PREDICT v2.1, 1697 patients (47.3%) were marked as clinical low risk and 1888 (52.7%) patients as clinical high risk.

If no SLNB would have been performed in our study population, this would have resulted in a changed pathological lymph node status from pN + to pNx in 722 of 3585 patients (20.1%). Based on the guideline of 2020, the chemotherapy recommendation changed to no indication in 475 of 1354 clinical high-risk patients (35.1%), which is 13.2% of the total study population. Based on PREDICT v2.1, the chemotherapy recommendation changed to no indication in 412 of 1,888 patients (21.8%), which is 11.4% of the total study population.

### Gene expression profile outcome

The 70-gene signature test (Mammaprint®) was used in 3,409 (95.1%) and the 21-gene Oncotype DX Breast Recurrence Score® in 176 (4.9%) patients. There were no patients who underwent both gene expression tests. A total of 1266 patients (36.5%) had a genomic high risk, and 2319 patients (64.7%) had a genomic low or intermediate risk. Patients with a genomic high risk (*n* = 1266) more often had NST (89.9% versus 84.4%, *p* < 0.001), T2 tumors (22.5% vs 19.1%, *p* = 0.053), grade III tumors (31.0% versus 6.8%, *p* < 0.001), and pN0 disease (83.8% versus 77.7%, *p* < 0.001) and were more often postmenopausal (66.6% versus 61.5, *p* = 0.033).

Of the study population, 758 (21.1%) were 50 years or younger, of whom 269 had a genomic high risk, and 489 had a genomic low or intermediate risk.

### Clinical risk compared to genomic risk

Using the guideline of 2020, we identified 1354 patients as clinical high risk of whom 652 (48.2%) had genomic high risk.

Using PREDICT v2.1, we identified 1,888 patients as clinical high risk of whom 848 (44,9%) had genomic high risk. More details regarding clinical risk compared to genomic risk scores are provided in Tables 2 and 3.

**Table 2:** The distribution of patients classified according to clinical risk by the guideline of 2020 compared to genomic risk by Mammaprint® or 21-gene Oncotype DX Breast Recurrence Score®

		Clinical risk, n (%)	
		Low	High
Genomic risk, n (%)	Low	1617 (72.5)	702 (51.8)
	High	614 (27.5)	652 (48.2)
	Total	2231 (100)	1354 (100)

**Table 3:** The distribution of patients classified according to clinical risk by PREDICT v2.1 compared to genomic risk by Mammaprint® or 21-gene Oncotype DX Breast Recurrence Score®

		Clinical risk, n (%)	
		Low	High
Genomic risk, n (%)	Low	1279 (75.4)	1040 (55.1)
	High	418 (24.6)	848 (44.9)
	Total	1697 (100)	1888 (100)

Of the 758 patients aged 50 or younger, 285 had a clinical high risk, which is 7.9% of the total population. Nowadays, this group is considered to be of high risk, despite the genomic outcome.

### Risk of chemotherapy undertreatment in case of no SLNB information

Of the 475 patients with a changed recommendation to no chemotherapy indication in case of no SLNB information based on the guideline of 2020, 126 patients (26.5%) had genomic high risk. Of the 412 patients with a changed recommendation to no chemotherapy indication in case of no SLNB information based on PREDICT v2.1, 82 patients (19.9%) had genomic high risk. Extrapolated to the whole study group, this concerns 3.5% (126 of 3585 patients) and 2.3% (82 of 3585 patients) of all patients, respectively. Consequently, this results in the number of GEPs needed to identify one patient at risk for systemic undertreatment in case SLNB is omitted of 28 in comparison to the guideline of 2020 (1/0.0351), and 44 in comparison to PREDICT v2.1 (1/0.0229).

## Discussion

Three randomized controlled BOOG 2013-08, SOUND, and INSEMA trials investigate the safety on omission of SLNB in cT1-2N0 breast cancer patients treated with BCT [1–3]. The SOUND trial recently showed positive safety results when SLNB is omitted in cT1N0 breast cancer [4], and the INSEMA trial revealed favorable morbidity results for the no-SLNB group [5]. In case SLNB will be omitted in this patient population in daily practice, there is a potential risk for systemic undertreatment due to lack of knowledge on positive lymph node status. In the current study, we assessed the added value of GEPs in cT1-2N0 ER+ HER2− breast cancer patients aged < 70 years and treated with BCT, to address whether GEP would affect the adjuvant chemotherapy indication and decrease the risk for systemic undertreatment. Our study showed that missing positive pathological lymph node status in 20.1% of our population would have led to a changed recommendation for adjuvant chemotherapy to no indication in 35.1% and 21.8% clinical high-risk patients when using Dutch breast cancer guideline of 2020 and online prediction tool PREDICT v2.1, respectively. Though compared to the GEP results, only 3.5% of these clinical high-risk patients had a genomic high risk and would have missed their adjuvant chemotherapy indication based on the guideline of 2020 and thus be at risk for systemic undertreatment. In contrast, PREDICT v2.1 provided an even lower risk of 2.3% of clinical high-risk patients who had genomic high risk based on GEP outcome. Therefore, 28 to 44 GEPs are needed (based on guideline of 2020 or PREDICT v2.1, respectively) to identify one patient at risk for undertreatment, which is not expected to be clinically profitable or cost-effective. To put this in further perspective, the low percentage of patients (2.3–3.5%) in whom a chemotherapy indication is missed will yield an even much lower and probably not significant difference in regional recurrence and overall survival. Final results of the previously described randomized controlled trials will provide more insight in near future.

For a long time, pathological lymph node information was used as the most important clinicopathological indicator for the recommendation of adjuvant systemic therapy. Since trial results of ACOSOG Z0011 and IBCSG 23–01 were presented [16–20], the need for information on the exact pathological lymph node status is being questioned. Both studies showed that omitting completion axillary treatment in case of sentinel lymph node metastases in patients treated with BCT did not result in inferior survival or recurrence risk, despite nodal metastases in 27% of the patients in the completion axillary lymph node dissection (ALND) group in the Z0011 study. The majority of the patients in both trials received adjuvant systemic therapy. The percentage of patients receiving adjuvant systemic therapy did not differ between patients treated with SLNB alone (97% in both studies) and patients treated with completion ALND (96% in the Z0011 and 95% in the IBCSG 23–01 trial) [16–20]. Because patients were treated with systemic therapy regardless of the performed surgical procedure (SLNB vs. ALND), this resulted in controversy regarding the importance of pathologic lymph node status for proper adjuvant systemic therapy indications. The above-mentioned randomized controlled trials showed that the absence of a complete pathological nodal node status did not influence the recommendation for adjuvant systemic therapy, nor did the 5- and 10-year rates of recurrence and OS [16–20]. A cohort study of 303 cT1-2N0 breast cancer patients furthermore revealed that even the absence of pathological lymph node information hardly impacted adjuvant systemic therapy recommendation, comparing the Dutch breast cancer guideline of 2012 and the in that time frequently used online prediction tool Adjuvant! Online [21]. The recommendation for adjuvant systemic therapy changed to no indication in 3.6% of the patients using the guideline of 2012 and in 1.0% with Adjuvant! Online, when comparing patients’ true pathological lymph node status to unknown pathological lymph node status. These results as well show a more limited influence of pathological lymph node status than one might initially expect. Study results are nevertheless not fully comparable with current study by differences in clinical high-risk definition between guidelines, the use of Adjuvant! Online instead of PREDICT, and the focus on adjuvant systemic therapy instead of chemotherapy specifically.

PREDICT is an internationally available online prediction tool, composed of several clinicopathological features and was validated in a patient population with a large percentage of ER + Dutch breast cancer patients (96.5%), making it most applicable to our study population [6, 7, 22]. A Dutch validation study showed the accuracy of PREDICT; however, the 5- and 10-year OS rates must be interpreted carefully in certain subgroups (i.e., ER-negative patients, patients aged > 75 years, T3 tumors) [23]. In our study, all patients were ER-positive, aged < 70, and had cT1-2 tumors. Therefore, PREDICT was highly applicable to our population, increasing the generalizability and reliability of our study results.

The 70-gene signature test (Mammaprint®) and 21-gene OncotypeDX recurrence score® have been developed to improve the selection of patients who do not benefit from adjuvant chemotherapy, despite a clinical high risk based on, for instance, online prognostic models like PREDICT. Both GEPs were already validated for ER + HER- breast cancer and since recently for node-positive ER+ HER2− breast cancer [24]. Based on 21-gene Oncotype DX Breast Recurrence Score®, the TAILORx trial showed in 2018 that ER+ HER2– node-negative breast cancer patients with an intermediate recurrence score (11–25) have no benefit of adjuvant chemotherapy with endocrine therapy compared to patients treated with endocrine therapy only, in terms of disease-free survival and OS [11]. Later, the TAILORx trial presented a subgroup analysis which showed that breast cancer patients aged < 50 years with an intermediate recurrence score and a clinical high risk do benefit from chemotherapy [25]. Comparable to this TAILORx subgroup analysis, a MINDACT exploratory analysis by age also showed clinically relevant chemotherapy benefit in clinical high-risk women aged 50 years or younger with a genomic low risk based on the Mammaprint®. In our study, 758 patients (21.1%) were 50 years or younger, of whom 285 had a clinical high risk, which is 7.9% of the total population. In these patients, the absence of nodal status information is not likely to change chemotherapy indication; moreover, a GEP should no longer be applied.

The Dutch breast cancer guideline of 2020 suggests using a GEP only in case controversy exists regarding the benefit of adjuvant chemotherapy based on clinicopathological factors in ER + breast cancer [13]. For our study, we included all patients in whom GEP was applied in the period of 2011 to 2017. In line with the guideline valid in that time period, the number of patients who were considered to be clinical high risk was much higher: 93.8% versus only 37.8% of the patients with today’s guideline. So, for 56.0% of the patients, the clinical risk would have changed to low risk, without GEP and with no chemotherapy indication. Despite the limited indication for GEPs now, the percentage of patients who would have missed their indication for adjuvant chemotherapy based on the guideline is low (3.5%). PREDICT v2.1 provides the lowest risk for systemic undertreatment (2.3%) and is currently often used in the Netherlands in addition to the guideline. Thus, this clinical and genomic high-risk group represents only a very small proportion of the population, making the risk of systemic undertreatment for the entire population very small. These low risks and the consequently high amount of GEPs (28–44) needed to identify one patient at risk of chemotherapy undertreatment are not expected to be clinically profitable or cost-effective. Nevertheless, the advantages of SLNB omission and the disadvantage of missing SLNB information must be carefully weighed against one another in shared decision-making with the patient.

## Conclusions

This study showed that information on pathological positive lymph node disease will be absent in 20.1% in case the SLNB is omitted in cT1-2N0 ER + HER- breast cancer patients aged younger than 70. According to the Dutch breast cancer guideline of 2020 and PREDICT v2.1, omission of SLNB will result in a very low risk of systemic undertreatment of 3.5% and 2.3%, respectively. The number of GEPs needed to identify one patient at risk for undertreatment does not justify its standard use. Final results of the randomized controlled trials on omission of SLNB will conclude on the safety regarding overall survival and recurrence risk.

## Data Availability

The datasets generated and analyzed during the current study are retrieved from the Netherlands Cancer Registry and are not publicly available due to its proprietary nature.
